# Facilitators and barriers for tuberculosis preventive treatment among patients with latent tuberculosis infection: a qualitative study

**DOI:** 10.1186/s12879-023-08612-2

**Published:** 2023-09-22

**Authors:** Anusha Manoharan, H. Siti Nur Farhana, K. Manimaran, Ee Ming Khoo, Wen Ming Koh

**Affiliations:** 1 Bandar Botanic Health Clinic, Bandar Botanic, Klang, Selangor 42000 Malaysia; 2 Institute for Health Behavioural Research, National Institutes of Health, Ministry of Health Malaysia, Block B3, Kompleks NIH, No 1, Jalan Setia Murni U13/52, Seksyen U13, Setia Alam, Shah Alam, Selangor 40170 Malaysia; 3 Department of Primary Care Medicine, Faculty of Medicine, Universiti Malaya, Kuala Lumpur, 50603 Malaysia; 4 Rawang Health Clinic, Jalan Rawang Perdana, Taman Rawang Perdana, Rawang, Selangor 48000 Malaysia

**Keywords:** Treatment decisions, Primary care, Qualitative study, Views, Latent tuberculosis, Facilitators, Barriers

## Abstract

**Background:**

Various factors influence tuberculosis preventive treatment (TPT) decisions thus it is important to understand the health beliefs and concerns of patients before starting TPT to ensure treatment compliance. This study aims to explore facilitators and barriers for TPT among patients diagnosed with Latent Tuberculosis infection (LTBI) attending six primary healthcare clinics in Selangor, Malaysia.

**Method:**

In-depth interviews were conducted face-to-face or via telephone among patients with a clinical diagnosis of LTBI using a semi-structured topic guide developed based on the common-sense model of self-regulation and literature review. Audio recordings of interviews were transcribed verbatim and analysed thematically.

**Results:**

We conducted 26 In-depth interviews; Good knowledge of active tuberculosis (TB) and its associated complications, including the perceived seriousness and transmissibility of active TB, facilitates treatment. LTBI is viewed as a concern when immune status is compromised, thus fostering TPT. However, optimal health is a barrier for TPT. Owing to the lack of knowledge, patients rely on healthcare practitioners (HCPs) to determine their treatment paths. HCPs possessing comprehensive knowledge play a role in facilitating TPT whereas barriers to TPT encompass misinterpretation of tuberculin skin test (TST), inadequate explanation of TST, and apprehensions about potential medication side effects.

**Conclusions:**

Knowledge of LTBI can influence TPT uptake and patients often entrust their HCPs for treatment decisions. Improving knowledge of LTBI both among patients and HCPs can lead to more effective doctor-patient consultation and consequently boost the acceptance of TPT. Quality assurance should be enhanced to ensure the effective usage of TST as a screening tool.

## Introduction

Tuberculosis (TB) is the second cause of mortality from a single infective agent, Mycobacterium tuberculosis, after COVID-19 above HIV and AIDS [1]. The 2021 Global Tuberculosis Report estimates 9.9 million people were infected with TB in 2020 which is equivalent to 127 cases per 100 thousand population [1]. Close to half of those cases (43%) occurred in South East Asia (SEA) region, followed by the Africa region (25%) and 18% in the Western Pacific region [1]. In the Western Pacific region, the incidence of TB is at 79 cases per 100 thousand population with an estimated 70,200 deaths occurring in 2020 [2]. Overall, there was a reduction in the annual TB incidence rate of 1% and death rate of 3.4% in the region, however, we are still far from achieving our target of End TB strategy in 2030 [2].

Although many individuals are infected with Mycobacterium tuberculosis, only 10% manifest as an active disease while the remaining persist in a latent phase, resulting in a large reservoir of individuals with Latent Tuberculosis Infection (LTBI) [3]. LTBI can be diagnosed by a positive Tuberculin Skin test or positive Interferon Gamma Release Assay (IGRA). A positive Tuberculin skin test cutoff value varies across different groups. A Tuberculin Skin test of 10 mm or more is considered positive in high-risk individuals and children except people living with HIV, organ transplant recipients, and people on immunosuppressive therapy [4, 5]. Various guidelines recommend treating LTBI in high-risk populations that include children less than 5 years of age, people living with Human immunodeficiency virus, recent close contacts of less than 2 years, organ transplant recipients, and those on immunosuppressive drugs [4]. Worldwide, the number of patients provided with Tuberculosis preventive treatment (TPT) has increased from 1 million in 2015 to 3.6 million in 2019, however in 2020 there was a reduction to 2.8 million possibly due to the disruption of health service as a result of the COVID19 pandemic [1]. Most successful group to receive TPT to date were the people living with HIV, the annual number increased from a mere 30,0000 in 2005 to 2.3 million in 2020 globally [1]. Malaysia is an intermediate TB burden country by WHO [6], with an incidence of 72.57 cases per 100,000 population in 2020, and a mortality rate of 7.12% [7]. As part of the WHO End TB strategy, the Ministry of Health (MOH) in Malaysia aims to eliminate TB by the year 2035 through various policies and programs [8]. The national strategic plan to treat LTBI in Malaysia began in September 2020 with the free availability of TPT at public healthcare clinics aimed at reducing the progression of LTBI among high-risk individuals to active TB [9]. Several steps have been taken to improve the management of LTBI including the introduction of IGRA testing options, and shorter treatment regimens compared to the current 6-month treatment of Isoniazid [10–12]. However current data from the MOH has shown a mere 50% uptake of TPT [13]. Barriers to treatment uptake include fear of treatment side effects, long treatment durations, access problems due to social circumstances, and poor doctor-patient relationship [14, 15]. It can be challenging to initiate TPT in asymptomatic individuals which requires an understanding of their health beliefs and concerns to ensure compliance in completing the treatment [16]. We aimed to explore facilitators and barriers that may influence patients’ decision to receive (or not) TPT. We hope the findings from this study can improve the management of LTBI in primary health care clinics.

## Methods

### Study design

Data collection for this qualitative study was conducted from July 2020 to December 2020.

### Study setting and population

Participants were purposively recruited based on age, ethnicity, and education level. Participants aged 18 years and above with a current or history of LTBI (positive Tuberculin Skin Test (TST) or IGRA and negative chest X-ray finding) in selected primary health care clinics in Petaling Jaya District, Selangor, Malaysia were included in the study. Patients identified with LTBI through close contact screening of active TB cases received ongoing care at a specialized LTBI clinic. Selangor is densely populated with a population from varied socioeconomic backgrounds.

### Data collection

Doctors in charge of the LTBI Clinic were approached and explained about the study. All patients attending the LTBI clinic were approached and offered the invitation to participate in this study by the doctors in charge. Details of participants who were keen to participate were informed to the researcher. The participants were approached and explained about the study once again by the researchers. Informed consent was obtained after clarification of the study objectives and activities. Consented participants were provided with an agreed time for interview according to their convenience. In-depth interviews were conducted face-to-face which was later converted to phone calls with audio recording due to the COVID-19 pandemic Movement Control Order (MCO) imposed by the government. Face-to-face in-depth interviews (IDI) were conducted in private consultation rooms to ensure confidentiality. Before the interview, written informed consent was re-explained and obtained from all the participants. For phone interviews, participant information sheets and consent forms were emailed to the participants who had agreed to participate following the informed consent explained over the phone to the participants. Written informed consent to participate in this study was obtained before the interview appointments. All participants were informed of the confidentiality and anonymity of data and that they could withdraw anytime from the study. Interviews were conducted in English or Malay (the national language) and lasted 30 to 60 min by WMK, AM, and SNFH who were fluent in both languages. Individuals were contacted by phone to seek clarification in case any further information was required.

**Fig. 1:**
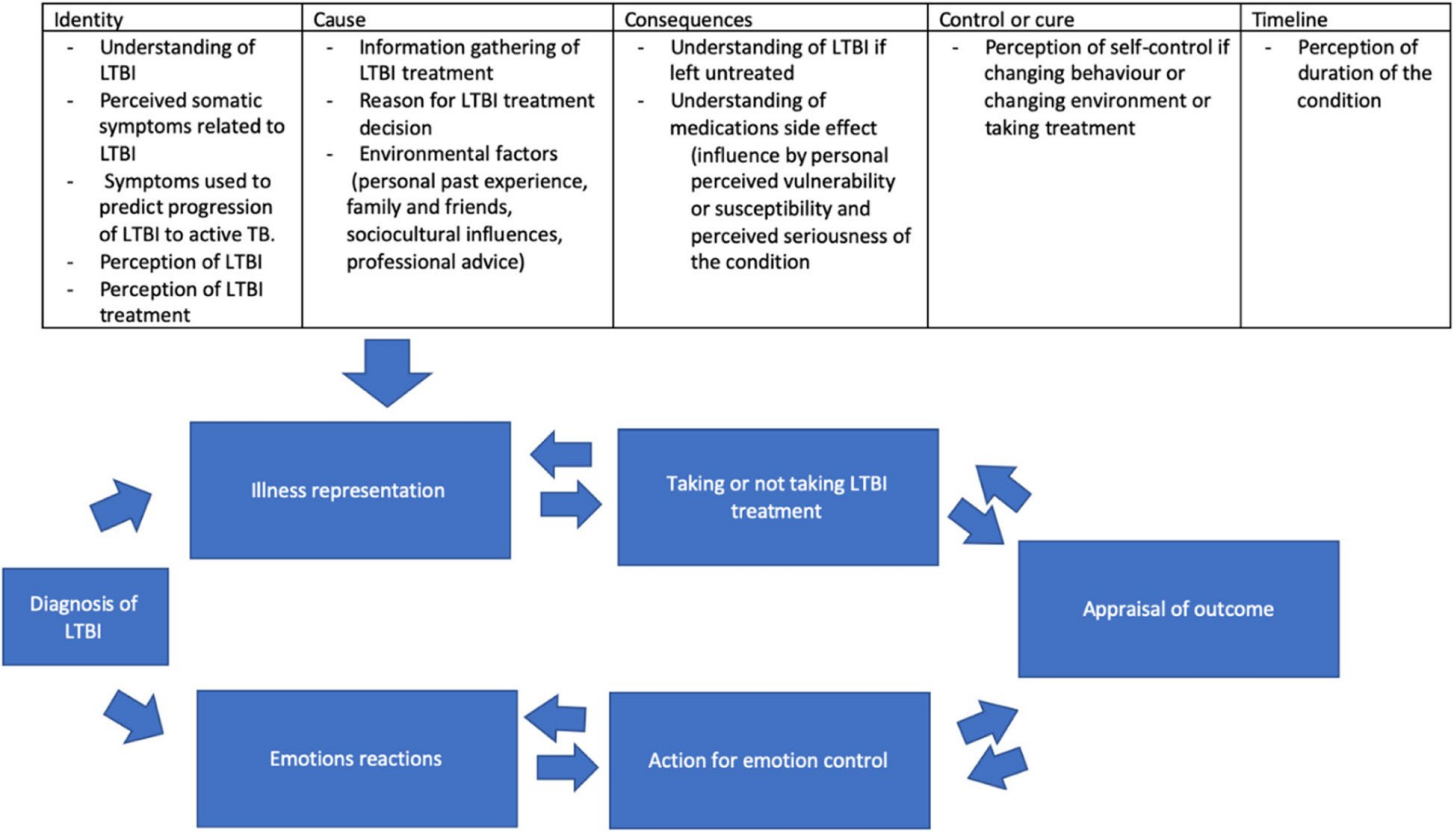
Theoretical framework for developing the topic guide

A semi-structured topic guide, which was developed from the Common Sense Model of Self-Regulation (CSM-SR) [17] and literature review was used in all of the interviews. CMS-SR was selected as it was one of the theoretical models that are frequently used in studies related to preventive medicine and medication adherence [18–20]. CSM-SR is a theoretical framework in which an individual perceives a health threat, which then develops two parallel yet interrelated cognitive and emotional responses towards the health threat with continuous feedback on the response and health threat [17]. Construction of the topic guide based on CSM-SR is represented in Fig. 1 below.

### Data analysis

All interview sessions were audio recorded and transcribed verbatim. The transcripts were checked for accuracy before analysis. Non-English language transcripts were interpreted in their originality. English language was used as the language for coding and analysis. All transcripts were entered in NVIVO version 12. WMK, AM, and SNFH familiarized themselves with the transcripts after each interview. Each researcher identified keywords or phrases from each transcript. Transcripts were coded independently. The initial coding frame was developed after the sixth interview and a discussion was done among all research team members (WMK, AM, SNFH, KEM, MK) on the coding framework. Any discrepancies were resolved during discussion and a consensus was reached for the coding framework. This coding framework was used to code the remaining transcripts after each interview. New keywords or phrases were identified and added to the coding framework after discussions with the research team. Saturation for the coding framework was achieved at the 23^rd^ interview and an additional three interviews were done to confirm data saturation. The codes were then arranged to form themes. Relationships between themes were connected to form a model. Non-English quotes selected for this paper were translated by a certified translator and checked by the interviewers to ensure no meaning was lost during translation.

### Research team & reflexivity

The audit trail was kept through journaling and discussion minutes by the research teams. WMK, AM, and SNFH were involved in participant interviews. WMK and AM are primary care physicians working in urban public healthcare clinics in Selangor and have experience conducting qualitative research in the past. SNFH is a health education officer working in a research institute in Malaysia with extensive qualitative research experience. All three researchers had no connection to the research participants before the study commencement. During the interviews, all three researchers identified themselves as research team members and explained the details of the study. The occupations of the research team members were not disclosed to the participants during all the interview sessions. During data analysis, WMK and AM used a field journal to remind themselves of their role as a researcher and not a clinician, while SNFH provided non-biased input. All research team members were trained and have conducted multiple qualitative research studies before this.

### Ethical approval and data protection

This study was conducted in accordance with the Declaration of Helsinki. Ethical approval was obtained from the Medical Research & Ethics Committee (MREC), Malaysia: [NMRR-19 3114-50974], KKM/ NIHSEC/P19-2683(11), before the research commencement. An amendment ethical approval was obtained in April 2021 from the Medical Research & Ethics Committee (MREC), Malaysia: NMRR-19-3114-50974 (IIR), KKM/NIHSEC/ P19-2683 before the change of data collection methods where face-to-face interviews were converted to phone interviews due to the movement control orders because of COVID 19 pandemic. The electronic consent form and phone interview data collection method were approved by the Medical Research & Ethics Committee in the amendment ethical approval NMRR-19-3114-50974 (IIR), KKM/NIHSEC/ P19-2683. All identifiers were removed and participants’ identities were anonymized during transcription. Data were stored in an encrypted and password-protected drive and are only accessible by the research team. Audio recordings were deleted after transcription and data checking were completed.

## Results

**Fig. 2:**
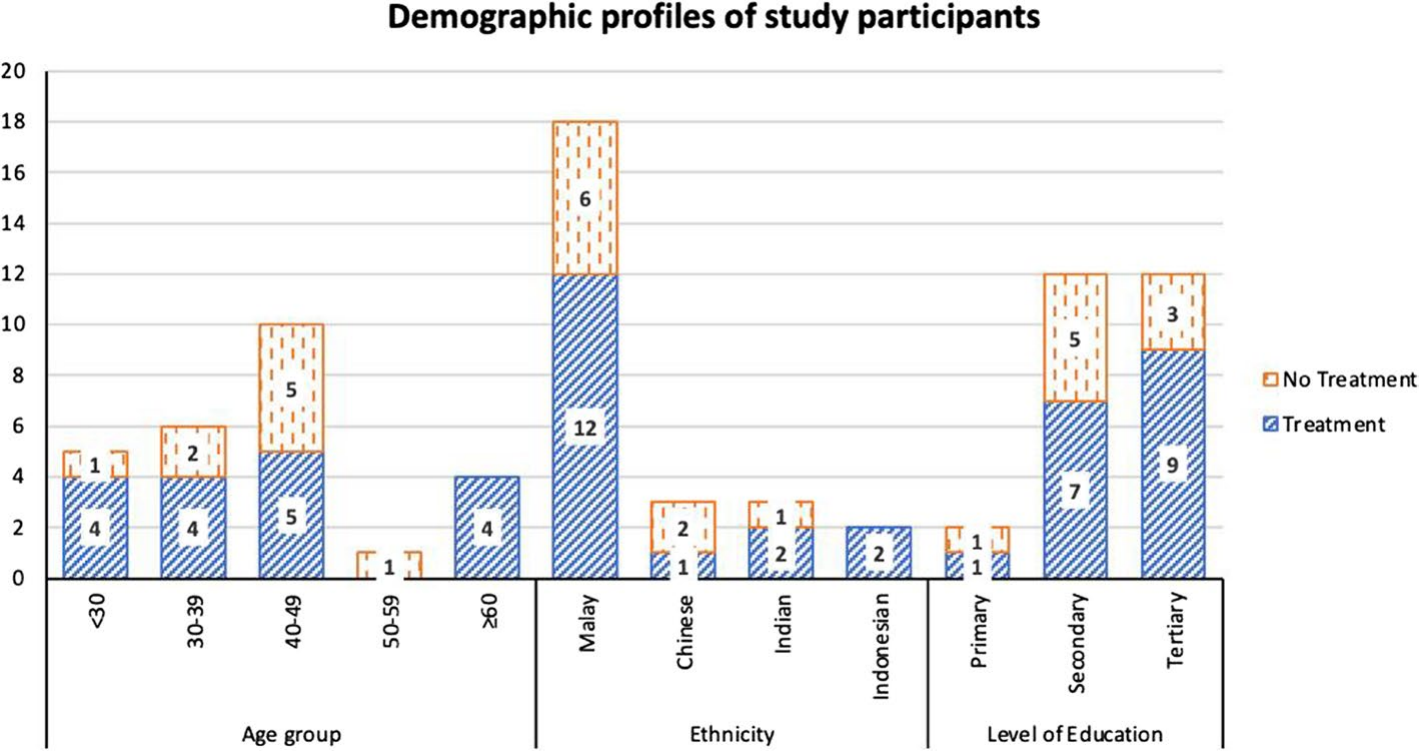
Demographic profiles of study participants

A total of 26 IDI was conducted, of which 17 accepted treatment and 9 refused. Participants’ ages ranged between 20 to 72 years old. There were 19 females and 7 males, 18 Malays, 3 Chinese, 3 Indians, and 2 Indonesians. 24 participants had completed secondary or tertiary education. Figure 2 describes the demographic profiles of the participants.

**Fig. 3:**
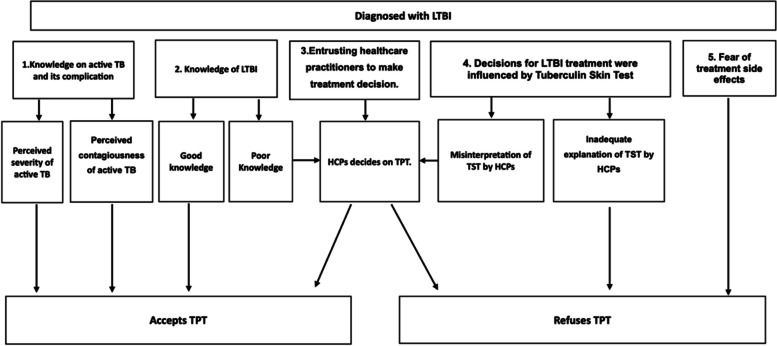
Facilitators and barriers that influence the decision for TPT

Five themes emerged that influenced patients’ decision to receive or refuse LTBI treatment. For a better understanding, themes have been characterized based on the facilitators and barriers that influence decision for TPT: (Fig. 3).

### Theme 1: Knowledge of active TB and its complications

#### Perceived severity of active TB

Participants had good knowledge of the symptoms of active TB and its complications. Their knowledge of active TB came from the experience of family and friends who had active TB. This knowledge of active TB served as a facilitator among participants for TPT uptake.

*“Like my dad, he had TB brain, but I still don’t know what kind of TB I am having… but for prevention, received treatment lah (exclamation) I know active TB…*

*(P15, 31 years old, received treatment)*

*“I have decided to receive treatment because my whole family such as my grandfather had TB. And maybe my uncles too, I cannot confirm they have TB but looking at their thin bodies and cough, maybe they have la, but they are villagers and did not go for treatment. So, because I had experience seeing this that is why I felt I should come for treatment.”*

*(P7, 69 years old, received treatment)*

#### Perceived contagiousness of active TB

Participants’ decision to take TPT was also made on their good knowledge of contagiousness and infectivity of active TB and the fear of spreading active TB to family and friends.

*“I don’t want to be like those who had TB (active). I took medicine at that time, I don’t want to spread it to others, if others had it, I would feel bad.”*

*(P10, 49 years old, received treatment)*

One participant feared reinfecting her husband although her husband has completed treatment for active TB.

*“I took the treatment … because I am afraid, I will spread it (active TB) to my husband, though he has taken medications (active TB medications), I am still afraid…”*

*(P20, 34 years old, received treatment)*

### Theme 2: Knowledge of LTBI

Knowledge of LTBI can be a facilitator and barrier to TPT. Some participants perceived their current healthy status as a protective factor against active TB as a barrier to LTBI treatment.

*“Ha, my friend who had ( active) TB completed treatment, I am not coughing, I am healthy. The inactive TB is like those with strong antibodies won’t get it, insha’Allah (if Allah wills it) … people with illness easier to get it”.*

*(P23, 44 years old not receiving treatment)*

Some understood LTBI as an immune-related condition that would be activated when one’s immune system is low which was a facilitator for TPT.

*“Because I had decided to take (the medications), because… because I know my condition, like easy to be exposed to other diseases because my antibody is low. So, When the doctor asked me to take (the medications), I just agreed lah (exclamation) …”*

*(P25, 47 years old, received treatment)*

### Theme 3: Entrusting healthcare practitioners to make treatment decisions

Due to the poor knowledge of LTBI, participants entrusted their HCP to decide on their treatment options.

*“Ha, I know I had TB, but I don’t know whether it’s active or not active, I just take whatever they say, because I have no knowledge!”*

(*P5, 49 years old, received treatment.)*

*“Because I heard latent TB does not infect anybody, so I didn’t take…He (the doctor) asked me… ‘You take (treatment)?’, I said, ‘No’ then he gave me advice. ‘If you don’t take this one, maybe, next time when your immune system is weak, the TB may become active…they now sleeping’ so best, you must take 6 months to finish this. Then, after that, only I started the 6 months (treatment)”*

*(P2, 72 years old, received treatment)*

A participant expressed that she had the intention to take TPT if necessary, however, she entrusted the HCP to decide on her treatment.

*“I was ready for the treatment, but if he (the doctor) said take, I will take. But he (the doctor) said no need for treatment as my chest X-ray was normal. So, I did not take the treatment and I was not stressed.”*

*(P25, 47 years old, did not receive treatment)*

### Theme 4: Decisions for LTBI treatment were influenced by Tuberculin Skin Test

The Tuberculin Skin Test was used as the primary screening test for LTBI in all the study participants.

#### Inadequate explanation of Tuberculin Skin Test by healthcare practitioners

Participants were not provided adequate information regarding the indication and interpretation of TST in the diagnosis of LTBI which resulted in one participant believing Tuberculin Skin Test as a treatment for LTBI.

“Doing this test (Tuberculin Skin Test), I don’t know what they do… I don’t know… he (doctor) asked me to get the injection, I just have it…”

(P13, 45 years old, not receiving treatment.)

*“When they did the injection (TST), they said they had injected the medications, so, it is impossible for it (LTBI) to get worse isn’t it, they said like this … (Tuberculin skin test) is to kill the (TB) bacteria…”*

*(P14, 38 years old, not receiving treatment)*

#### Misinterpretation of TST by HCPs

Some HCPs failed to diagnose LTBI with a positive TST which resulted in participants being reassured that the positive TST was normal and did not require any treatment. This led to participants not taking TPT.

*“We have seen the doctor; the doctor said the size (15mm after TST) is like nothing. He said, ‘This is ok’ The doctor said. After this, the doctor asked to come back for a follow-up. We do not think about it anymore after that.”*

*(P23, 44 years old, not receiving treatment.)*

Some participants doubted the diagnosis of LTBI due to the discrepancies in TST results interpretation by different categories of HCP resulting in TPT refusal.

*“But he (the doctor) said I already got it, after this I want to go to private to check my blood (IGRA)… because I am also not sure, The nurse said I do not have… my son and brother have the same size (Tuberculin skin test), but my son is positive, but my brother is not, I am also surprised (laughs)…”*

*(P21, 40 years old, not receiving treatment.)*

### Theme 5: Fear of treatment side effects

Fear of treatment side effects influences the decision of TPT.

*“Because I feel I already have many medical illnesses. So, if I want to take the medicine, I am afraid also, to take the medicine. Moreover, they (doctors) said there is side effect, right”.*

*(P14, 38 years old, not receiving treatment)*

## Discussions

Participants perceived severity and contagiousness of TB allowed them to perceive the benefit for TPT and influenced their behaviour to receive treatment which can be explained by the Health Belief Model (HBM) [21]. Similar findings were found among household close contacts and those diagnosed with LTBI who perceived the seriousness, susceptibility, and benefit of TPT and practiced preventive measures to reduce their risk of contracting active TB [22, 23]. HBM can also explain the poor uptake of TPT among participants with poor knowledge of LTBI as they were not able to perceive their susceptibility to contracting active TB or the perceived severity or risk of their diagnosis resulting in TPT refusal. HBM-based education to increase awareness and knowledge of LTBI in the community will be the key to facilitating TPT uptake [24].

According to the CSM-SR model, emotional response plays a role in treatment decisions [17]. Knowledge of active TB and its complications as well as knowledge of LTBI triggers a fear response that leads to proactive behaviour. This proactive behavior facilitates the acceptance of TPT. Conversely, knowledge of TPT and its side effects evokes a fear of TPT which in turn is a barrier to TPT uptake [17, 25]. Leventhal et al. outlined that the decision-making process on TPT is influenced by sociocultural context and past experiences [17], particularly those of loved ones that have dealt with active TB. These shared experience, coupled with emotions such as fear of TB threat plays a pivotal role in facilitating participants’ acceptance of TPT [25].

Participants had good knowledge of active TB and its complications with varied knowledge of LTBI. Having a good understanding of active TB leads to heightened awareness of active TB complications, prompting strategies such as taking TPT to prevent having an active TB. These findings correlate with the results from a study conducted in Malaysia where TB contacts who have good knowledge have a constructive attitude towards screening and TPT [26]. Variable knowledge of LTBI could be a facilitator or barrier to LTBI treatment. LTBI is understood as an immune-mediated condition where participants with multiple co-morbidity perceive themselves in a weaker “immune” condition putting them at risk of active TB disease. This understanding prompts the need for TPT. On the other hand, LTBI was understood as an asymptomatic condition and a resemblance to a healthy condition was a barrier to treatment [27]. These findings highlight the importance of counseling by HCPs during the initial care process of a patient diagnosed with LTBI. Providing patient education and a detailed explanation of the LTBI cascade of care will improve the knowledge of patients on the importance of follow-up and receiving TPT [23]. A well-informed patient is more likely to perceive the potential risk associated with untreated LTBI and as a result, may be more motivated to undergo treatment for LTBI to mitigate these risks [23, 28].

Participants delegate the decision-making regarding TPT to HCPs, either facilitating or impeding TPT uptake. Similar findings were noted from a study among Chinese immigrants in Canada where patient and HCP trust is a barrier or facilitator for TPT [27]. Patient who trusts their HCP with good skills and ethics takes TPT while the misconception of doctors using drugs as the test is a barrier to TPT [27]. This differs from the norm in many developed countries where patients are involved in their health and treatment choices [29]. Reasons for this could be due to the lack of LTBI knowledge among patients and HCPs which is crucial in the treatment decision-making process [30]. HCPs that are equipped with good knowledge of LTBI facilitate TPT and vice versa. Therefore, HCPs need to be equipped with knowledge of LTBI to provide accurate and adequate consultations to patients to achieve well-informed TPT decisions [27]. Through adequate and accurate LTBI knowledge transfer across effective communications and good relationships between patients and HCPs facilitates TPT [31, 32]. This could be further enhanced with the use of patient decision aid on LTBI to facilitate a conversation and better understanding between patients and their HCP to assist in arriving at a treatment decision [27, 33].

TST is a cost-effective screening test that can be performed by any healthcare worker who has received training. Our findings reiterate the challenges when using TST in particular subjective evaluation of the skin reaction with intra and inter-observer errors [34]. Different HCPs evaluating the TST results during each cascade of care increases the possibility of misinterpretation and TST results. Conflicting answers by HCPs can result in confusion and doubts about the TST test and treatment refusal by patients for LTBI. The usage of digital tools like smartphones with applications to read TST can allow direct observation for ongoing monitoring and quality assurance which can be difficult when TST is performed by different HCPs and read by different HCPs. Photos can be taken by patients should there be adverse reactions [34] or doubts and accurate interpretation can be done on the day of evaluation by the HCPs [35]. IGRA should be considered as a definitive diagnostic tool among patients who may have doubts and uncertainties about diagnosis with TST and refuse treatment. This could be valuable to convince patients on the treatment should their IGRA test be positive and in those whose TST was a false positive result they can safely be counselled not for treatment [34].

## Strengths and limitation

We conducted the study among an intermediate TB burden country with diverse ethnicity and sociodemographic profiles, informing views from diverse cultural backgrounds. Existing qualitative data have mainly focused on immigrant populations in nations with low tuberculosis prevalence. Future studies can focus on the development of a patient-centered training module and patient decision aids to increase treatment uptake through shared decision-making.

## Conclusion

Patients’ TPT decision was influenced by poor disease awareness and knowledge, and a reliance on the HCP to make their treatment decision. HCPs need to equip themselves with adequate knowledge and consultation skills to assist and guide their patients in their decision-making process. There is a need to re-evaluate existing LTBI screening and treatment programs to address these gaps.

## Recommendation

The current recommendation based on Malaysian CPG [5] includes systematically testing using TST or IGRA and treating at-risk groups for example households and close contact with bacteriologically confirmed PTB, People living with HIV, receiving dialysis, and preparing for organ or haematological transplant. Uncertainties in the decision for treatment can occur with the usage of TST and can result in uncertainties in diagnosis. We recommend in high-risk groups a definitive diagnostic test such as IGRA be offered to eliminate doubt and increase uptake. A better understanding of LTBI enhances treatment uptake therefore there is a need to provide accurate information on LTBI and its treatment via patient education and community empowerment.

## Data Availability

The findings of this study are shared as anonymized quotes in this paper. Additional data required can be requested through the corresponding author with reasonable justification.

## Abbreviations

TPT: Tuberculosis preventive treatment
LTBI: Latent Tuberculosis infection
HCPs: Health Care Practitioners
HBM: Health Believe Model
TB: Tuberculosis
SEA: Southeast Asia
IGRA: Interferon Gamma Release Assay
MOH: Ministry of Health
TST: Tuberculin Skin Test
MCO: Movement Control Order
IDI: In-depth Interviews
CSM-SR: Common Sense Model of Self-Regulation
MREC: Medical Research & Ethics Committee
